# Psychological impact of COVID-19 on diabetes mellitus patients in Cape Coast, Ghana: a cross-sectional study

**DOI:** 10.11604/pamj.2021.40.76.26834

**Published:** 2021-10-05

**Authors:** Richard Kobina Dadzie Ephraim, Evans Duah, Charles Nkansah, Samuel Amoah, Emmanuel Fosu, Justice Afrifa, Felix Botchway, Perditer Okyere, Samuel Essien-Baidoo, Kofi Mensah, Dorcas Serwaa, Samuel Asamoah Sakyi, Prince Adoba, Linda Ahenkorah Fondjo, Jerry Paul Ninnoni, Yaa Boahemaa Gyasi Aderoju

**Affiliations:** 1 Department of Medical Laboratory Science, University of Cape Coast, Cape Coast, Ghana,; 2 Department of Epidemiology and Disease Control, School of Public Health, University of Ghana, Legon, Ghana,; 3 Dream Laboratory Consult, Cape Coast, Ghana,; 4 Department of Medical Diagnostics, Kwame University of Science and Technology, Kumasi, Ghana,; 5 University Health Services, University of Cape Coast, Cape Coast, Ghana,; 6 Department of Chemical Pathology, University of Ghana, Legon, Ghana,; 7 Department of Medicine, School of Medical Sciences, Kwame University of Science and Technology, Kumasi, Ghana,; 8 Department of Molecular Medicine, School of Medical Sciences Kwame University of Science and Technology, Kumasi, Ghana,; 9 Department of Obstetrics and Gynaecology, University of Ibadan, Ibadan, Nigeria,; 10 Trauma and Specialist Hospital, Ghana Health Service, Winneba, Ghana,; 11 School of Nursing, University of Cape Coast, Cape Coast, Ghana

**Keywords:** COVID-19, psychological Impact, diabetes mellitus, Cape Coast, Ghana

## Abstract

**Introduction:**

COVID-19 pandemic has had a greater psychological impact on patients with chronic ailments such as diabetes mellitus, tuberculosis, and HIV/AIDS compared to those without chronic conditions. We explored the psychological impacts of COVID-19 among people living with diabetes mellitus in Ghana.

**Methods:**

this study employed a hospital-based cross-sectional design involving 157 diabetes mellitus patients aged 20 years and above. We assessed diabetes distress by the seventeen-item diabetes stress (DDS17) scale and COVID-19 worries by 3 specific benchmarks: “worry about overly affected due to diabetes if infected with COVID-19”, “worry about people with diabetes characterized as a risk group” and “worry about not able to manage diabetes if infected with COVID-19”. A close-ended questionnaire was used in data collection.

**Results:**

of 157 diabetic patients interviewed, the majority had type 2 diabetes mellitus with known complications and only 42.7% were managing COVID-19 symptoms. The participants showed moderate to high level of COVID-19 specific worry, moderate fear of isolation, and low level of diabetes-associated distress. About 33.8% of the study population expressed a sense of worry towards the pandemic. The logistic regression showed that age, employment status, and presence of other chronic diseases were significantly associated with worries about being overly affected if infected with COVID-19 due to their diabetes status. Age and sex were associated with worries about people with diabetes being characterized as a risk group and age, sex and employment status were associated with participants who were worried about not being able to manage diabetes if infected with COVID-19.

**Conclusion:**

the general trend indicates a sense of worry among diabetes patients during the COVID-19 pandemic which is associated with poorer psychological health. Clients' education and counseling on COVID-19 are necessary to address some of their concerns to minimize the level of anxiety and emotional stress in these individuals.

## Introduction

COVID-19, with a global mortality rate of 3-4%, presents with mild to severe symptoms including sore throat, dry cough, runny nose, dysphagia and occasionally with diarrhoea and vomiting [1]. The aged, immunocompromised individuals and people with pre-existing diseases tend to suffer severe complications and have a higher mortality rate when they are exposed to SARS-CoV-2 [2].

Presently, there exists a vast array of information including unverified malicious information on social media and these can spread quickly and can misinform, cause fear and panic among citizens in countries with confirmed cases of the disease [3]. The associated psychological effects of the COVID-19 pandemic among people living in countries with diagnosed cases include anxiety, stress and depression [4-6]. Further, “quarantine” which was one of the main mechanisms used by most countries in combating community spread in itself has been associated with a myriad of psychological problems including confusion, anger as well as possible post-traumatic stress symptoms such as panic of being infected, dullness, frustrations, economic instability, stigma and insufficient supplies [5,7].

The fear of developing any of the long-term complications of diabetes mellitus such as organ failure, stroke, etc. negatively influences the quality of lives of affected individuals, as it may drastically reduce their life expectancies. Relative to the general population, about 40% of diabetic mellitus patients suffer diabetes stress and develop several psychological problems which could result in anxiety, depression, and the development of disturbed eating habits [8,9]. Studies have established that the COVID-19 pandemic has a greater psychological impact on patients with chronic ailments like diabetes mellitus and HIV/AIDS than on those without chronic conditions [10,11]. Consequently, the emotional impact of the COVID-19 pandemic may exacerbate the already existing concerns of psychological problems in diabetic patients [12], especially in a low-income country like Ghana with a 3% prevalence of diabetes [13].

Despite the significance of COVID-19 associated psychological implications among people with diabetes, no such data exist in Ghana. Consequently, the need to routinely assess and analyze data in this regard is of prime importance as this is likely to affect their overall quality of life and well-being as well as inform educators and counselors in the management of this disease. This cross-sectional study sought to explore the psychological impacts of COVID-19 among people living with diabetes mellitus in Ghana.

## Methods

**Study setting, design and participants:** this hospital-based cross-sectional study was conducted in Ghana from June to September, 2020. The study recruited 157 out of the 180 diabetes mellitus patients aged 20 years and above who were being managed at the diabetic clinics of two (2) health facilities in the Cape Coast District of Ghana; the Ewim Polyclinic and the University of Cape Coast Hospital. These health facilities serve over 20 settlements in the Cape Coast Metropolis in the central region of Ghana. We employed the convenience sampling technique. This permitted the inclusion of patients who were willing to share core information about their medical and psychological health amidst the COVID-19 pandemic and volunteered to participate in the survey. In all, 90 people were recruited from the Ewim Polyclinic and 67 from the University of Cape Coast Hospital.

### Sample size determination


$$n\geq \frac{P\left(1-P\right)Z^{2}{}_{\left(1-\alpha /2\right)}}{e^{2}}$$

where: n = sample size required; Z = Z score for 95% confidence interval = 1.96; P = estimated prevalence of diabetes mellitus = 6.5% [14]; e = margin of error (5% =0.05).

**Structure of the questionnaire and variables:** a close-ended structured questionnaire was adopted for this study. The questionnaire contained information on socio-demographic characteristics, clinical information, diabetes-specific social support, diabetes distress, modification in diabetes-specific behaviours and worries of diabetic patients about COVID-19. The socio-demographic characteristics and clinical information included age, sex, educational status, marital status, type of diabetes mellitus, management of complications and any other chronic disease among others. Issues concerning whether the families or participants themselves had COVID-19 manifestations or were confirmed with COVID-19, with or without hospital admission, were also captured.

Questions on diabetes-related social support from relatives, friends, colleagues at work, other people with diabetes and social media were also included. Diabetes distress was assessed by the seventeen-item diabetes stress (DDS17) scale. Changes in diabetes-related behaviours arising from the COVID-19 pandemic were assessed with a “yes/no” on a list of possible modifications in behaviours related to blood glucose checks, medication, diet, physical activity and other behavioural changes. Questions on COVID-19-specific worries consisted of one question assessing general issues relating to the COVID-19 pandemic and “yes/no” list of possible diabetes-related COVID-19 pandemic issues.

**Data collection:** we employed the convenience sampling method to obtain responses from participants in the hospital setting using a structured questionnaire administered via computer-assisted personal interviewing tablet by the research team whilst we adhered strictly to COVID-19 safety protocols from the Ghana Health Service. This sought to prevent the possible community transmission of COVID-19.

**Ethical approval:** the study was approved by the University of Cape Coast IRB. Written permission was sought from the administrators of the health facilities included in the study. Informed consent was sought from patients recruited for the study by reading out a confidentiality statement using the computer-assisted personal interviewing tablet. Only participants who agreed to participate were included in the study. The questionnaire did not collect names and address of participants to ensure privacy and patient’s confidentiality.

**Operational definitions:** the dependent variable was diabetes-associated worries related to the COVID-19 pandemic, namely: ‘worried about being overly affected due to diabetes if infected with COVID-19’, ‘worried about people with diabetes being characterized as a risky group’ and ‘worried about not being able to manage diabetes if infected with COVID-19’. The independent variables were socio-demographic and clinical factors and psychosocial and behavioural factors.

**Data analysis:** data were extracted using Microsoft Excel version 2016 and statistical analysis was run using StataCorp LLC’s Stata/IC version 16.0. Normality tests were conducted, and the findings revealed that normality rules were met, which is why mean ± SD were used to represent continuous data. Descriptive summary statistics frequencies, percentages and graphs were used appropriately. To establish the independent factors influencing diabetes-related worries to the COVID-19 pandemic among the participants, binary logistic regression analysis was performed with the model and statistical significance set at p<0.05 and 95% confidence level. All coefficients were reported as odds ratios (OR) with 95% confidence intervals (CI) and standard errors (SE).

## Results

**Figure 1:**
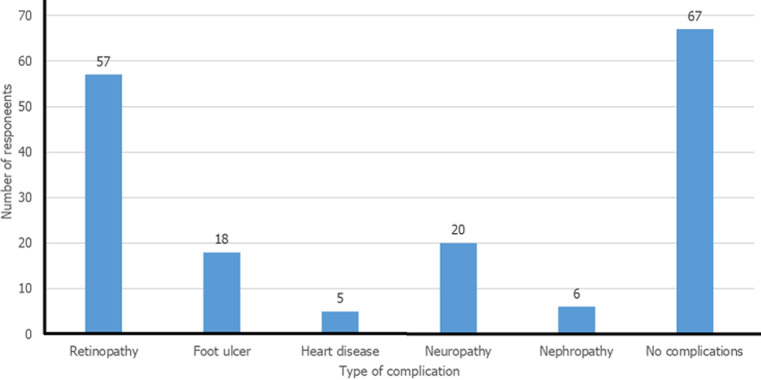
respondents managing diabetes mellitus complications

Among the 157 study participants, 68 (43.3%) were aged 40-59 years, 94 (59.9%) were females and 51 (32.5%) had attained basic school education. Regarding their employment status, 105 (66.9%) were employed and 6 (3.8%) had been laid-off temporary or permanently from employment. The majority, 120 (76.4%) were married and 63 (40.1%) had 1-3 children (Table 1). The majority of the participants (72.6%) had type 2 diabetes mellitus, 90 (57.3%) had known complications, 57 (36.3%) had retinopathy (Figure 1); with 32 (20.4%) having level 3 complications. More than half of the participants (54.8%) were comorbid with hypertension, 67 (42.7%) were managing COVID-19 symptoms and only 1.3% had relatives who had tested positive for COVID-19 at the time of collecting the data (Table 2). Figure 1 shows the distribution of diabetes mellitus-related complications recorded among respondents. The majority of the respondents reported complications of the eye (retinopathy) (57 out of the 157 respondents). The participants showed significantly moderate to high level of COVID-19 specific worry (mean score: 7.2 ± 0.25, 95% CI: (6.7-7.7)). Fear of isolation was moderate among the participants (mean score: 4.8 ± 0.28, 95% CI: (4.2-5.3)) and also a low level of diabetes-associated distress was observed within the studied population (mean score: 2.2 ± 0.09, 95% CI: (1.9-2.3)) (Table 3).

**Table 1:** general characteristics of study population

Variable	N=157 n (%)
**Age group (years)**	
20 - 39	24 (15.3)
40 - 59	68 (43.3)
≥60	65 (41.4)
**Sex**	
Male	63 (40.1)
Female	94 (59.9)
**Education status**	
No formal education	38 (24.2)
Basic	51 (32.5)
Secondary	22 (14.0)
Tertiary	46 (29.3)
**Employment status**	
Employed	105 (66.9)
Unemployed	17 (10.8)
Lay-off	6 (3.8)
Retired	29 (18.5)
**Marital status**	
Single	10 (6.4)
Married	120 (76.4)
Divorced	13 (8.3)
Widowed	14 (8.9)
**Number of children**	
None	13 (8.3)
1 - 3	63 (40.1)
4 - 6	60 (38.2)
>6	21 (13.4)

**Table 2:** distribution of clinical information among the study participants

Variable	N=157 n (%)
**Type of diabetes mellitus**	
Type 1	18 (11.5)
Type 2	114 (72.6)
Unknown	25 (15.9)
**Managing diabetes complication?**	
Yes	90 (57.3)
No complication	67 (42.7)
**Severity of complication**	
Level 1	7/90 (4.5)
Level 2	24/90 (15.3)
Level 3	32/90 (20.4)
Level 4	8/90 (5.1)
Level 5	19/90 (12.1)
**Any other chronic disease?**	
Arthritis	6 (3.8)
Gastric ulcer	5 (3.2)
Hypertension	86 (54.8)
Mental illness	6 (3.8)
No other chronic disease	54 (34.4)
**Are you managing any COVID-19 symptom?**	
Yes	67 (42.7)
No symptoms	90 (57.3)
**Have you or a relative tested positive to COVID-19?**	
Yes	2 (1.3)
No	155 (98.7)

**Table 3:** psychosocial factors of COVID-19 on the study participants

Variable (N=157)	Mean Score ± SD (CI)
COVID-19 specific worry, scale from 1 (low) to 10 (high)	7.2 ± 0.25 (6.7 - 7.7)
Fear of Isolation, scale from 1 (low) to 10 (high)	4.8 ± 0.28 (4.2 - 5.3)
Diabetes distress (DDS17 score)	2.2 ± 0.09 (1.9 - 2.3) ♠

♠ A mean item score of 3 or higher indicates moderate distress hence a level of distress worthy of attention

**Figure 2:**
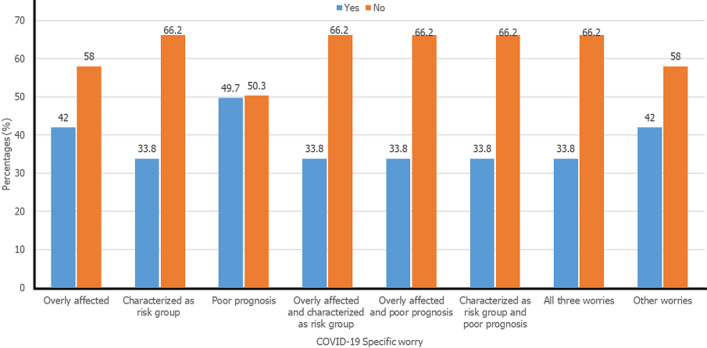
COVID-19 worries among respondents

The results indicate that a higher percentage of diabetic mellitus patients (79.6%) received moderate to high social support from their family, friends and close relatives. Also, 61.2% of them received moderate to high social support from diabetic team and an equal number of them (61.2%) received moderate to high social support from people living with diabetes mellitus (61.2%), followed by social media (56.7%) and people at workplace and schools (52.9%). Regarding behavioural changes, 130/157 (82.8%) were ‘more careful about taking medication than usual´, 66 (42.0%) ate less than usual and a minority, (31.2%) checked their blood glucose more than usual. In all, 150 (95.5%) of the respondents had no change in other behavioural activities (Table 4). Figure 2 gives the proportion of worried respondents according to the benchmarks employed. Out of the 157 respondents, 42% expressed worry “they would be overly affected if infected with coronavirus due to diabetes”, 33.8% worried “about people with diabetes being characterized as a risk group” whereas 49.7% worried about not being able to manage diabetes if infected with coronavirus. Equal distribution of worry was recorded among those who worried about any 2 or all 3 of the benchmarks (33.8%). However, 42% expressed no worry at all in any of the benchmarks.

**Table 4:** social support and behavioural change among the participants

Variable (n=157)	N (%)
**Moderate to high social support**	
Family, friends and close people	125 (79.6)
People at workplace or school	83 (52.9)
Diabetes care team	96 (61.2)
Other people living with diabetes	96 (61.2)
Social media	89 (56.7)
**Behaviour change (positive responses)**	
Check blood glucose more than usual	49 (31.2)
More careful about taking medications than usual	130 (82.8)
More exercise than usual	53 (33.8)
Less exercise than usual	65 (41.4)
Eat more than usual	51 (32.5)
Eat less than usual	66 (42.0)
**Other behavioural changes**	
No change	150 (95.5)
Other changes	7 (4.5)

Seven (7) independent variables were entered into the bivariate logistic regression to determine the factors associated with COVID-19-specific worries. Age, employment status, and presence of other chronic diseases were significantly associated with participants who ‘worried about being overly affected due to diabetes if infected with COVID-19´. Only age and sex were significantly associated with participants who “worried about people with diabetes being characterized as a risk group” and age, sex and employment status were significantly associated with participants who ‘worried about not being able to manage diabetes if infected with COVID-19´.

Diabetic patients aged between 40-59 years were less likely to be worried about being characterized as a risk group (OR=0.26, (95%CI=0.07-0.97), P=0.045) and not being able to manage diabetes if infected with COVID-19 (OR=0.33, 95%CI=0.12-0.94), P=0.038) compared with those aged between 20-39 years. Also, participants aged ≥60 years were less likely to worry about being overly affected due to diabetes (OR=0.24, (95% CI =0.08-0.72), P=0.011), being characterized as a risk group (OR=0.21, (95% CI 0.06,0.79), P=0.021) and not being able to manage diabetes if infected with COVID-19 (OR=0.24, (95%CI=0.08-0.68), P=0.007) compared to their 20-39 years´ counterparts.

Females were also more worried than men about people with diabetes mellitus being characterized as a risk group and not being able to manage diabetes if infected with COVID-19 (OR=2.21(95% CI=1.12, -4.34), P=0.022) and (OR=2.56(95% CI=1.33-4.95), P=0.005) respectively. People who had retired from active service were less likely to worry about being overly affected due to diabetes (OR=0.34(95% CI=0.14-0.79), P=0.013) and not being able to manage diabetes mellitus if infected with COVID-19 compared to those employed (OR=0.41(95% CI =0.17-0.98), P=0.045). Lastly, participants comorbid with arthritis were less likely to have worries about being overly affected by COVID-19 compared with people with no other chronic diseases (OR =0.09(95% CI =0.01-0.85), P=0.035) (Table 5).

**Table 5:** odds of experiencing COVID-19 worries according to socio-demographic and clinical factors

	Worried about being overly affected due to diabetes if infected with COVID-19		Worried about people with diabetes being characterized as a risk group		Worried about not being able to manage diabetes if infected with COVID-19	
Variable	OR (95% CI)	P-value	OR (95% CI)	P-value	OR (95% CI)	P-value
**Age group (years)**						
20 - 39	1		1		1	
40 - 59	0.39(0.13-1.19)	0.102	0.26(0.07-0.97)	0.045	0.33(0.12-0.94)	0.038
≥60	0.24(0.08-0.72)	0.011	0.21(0.06-0.79)	0.021	0.24(0.08-0.68)	0.007
**Sex**						
Male	1		1		1	
Female	1.82(0.95-3.49)	0.070	2.21(1.12-4.34)	0.022	2.56(1.33-4.95)	0.005
**Employment status**						
Employed	1		1		1	
Unemployed	2.08(0.64-6.83)	0.226	2.23(0.60-8.30)	0.230	1.67(0.57-4.84)	0.348
Layed-off	1.28(0.22-7.31)	0.780	2.39(0.27-21.30)	0.434	1.82(0.32-10.36)	0.501
Retired	0.34(0.14-0.79)	0.013	0.45(0.19-1.03)	0.059	0.41(0.17-0.98)	0.045
**Any other chronic disease?**						
Arthritis	0.09(0.01-0.85)	0.035	0.18(0.03-1.06)	0.058	0.17(0.02-1.58)	0.119
Gastric ulcer	0.69(0.11-4.51)	0.698	0.53(0.08-3.47)	0.504	0.22(0.02-2.06)	0.182
Hypertension	0.50(0.25-1.03)	0.060	0.59(0.28-1.25)	0.168	0.95(0.48-1.87)	0.874
Mental illness	2.29(0.25-21.20)	0.463	1.75(0.19-16.30)	0.623	0.86(0.16-4.66)	0.863
No other chronic disease	1		1		1	

As shown in Table 6, fear of isolation was associated with being worried about not being able to manage diabetes if infected with COVID-19. Participants who score 1-5 points on the scale of the fear of being isolated were 99% less likely to experience worries about not being able to manage diabetes if infected with COVID-19 compared with those who scored 6-10 on the same scale (OR=0.01, (95% CI=0.00-0.04), P=0.000). Individuals who received moderate to high social support from their family, friends and close relatives were less likely to be worried about being overly affected (OR=0.33, (95% CI=0.16-0.66), P=0.002) and about people with diabetes being characterized as a risk group (OR=0.38, (95% CI=0.18 -0.79), P=0.010) compared with those who received no social support. Also, diabetic patients who were cared for by other people living with diabetes had 75%, 67% and 50% less likelihood of ‘worry about being overly affected due to diabetes if infected with COVID-19´ (OR=0.25, (95% CI=0.12-0.52), P=0.000), ‘worry about people with diabetes being characterized as a risk group´ (OR=0.33, (95% CI=0.16-0.69), P=0.004) and ‘worry about not being able to manage diabetes if infected with COVID-19´ (OR=0.50, (95% CI=0.26 to 0.97), P=0.04) respectively compared with those who received no social support. Likewise, participants who obtained social support from social media had less likelihood of worrying about being overly affected due to diabetes if infected with COVID-19 (OR=0.24, (95% CI=0.12-0.48), P=0.000) and people with diabetes being characterized as a risk group (OR=0.38, (95% CI=0.19-0.77), P=0.008) relative to people who did not experience any form of social support. Lastly, those participants who had a greater likelihood of worrying about not being able to manage diabetes if infected with COVID-19 were less exercising compared with people who had no behavioural changes (OR=3.04, (95% CI =1.57-5.89), P=0.001).

**Table 6:** odds of experiencing COVID-19 worries according to psychosocial and behavioural factors

	Worried about being overly affected due to diabetes if infected with COVID-19		Worried about people with diabetes being characterized as a risk group		Worried about not being able to manage diabetes if infected with COVID-19	
Variable	OR (95% CI)	P-value	OR (95% CI)	P-value	OR (95% CI)	P-value
**Fear of isolation scale**						
1-5	0.02(0.01-0.08)	0.000	0.04(0.01-0.15)	0.000	0.01(0.00-0.04)	0.000
6-10	1		1		1	
**Social support (individual reference is: do not get support)**						
Family, friends and close people	0.56(0.24-1.28)	0.017	0.48(0.19-1.19)	0.116	0.63(0.29-1.39)	0.253
People at workplace or school	1.22(0.65-2.30)	0.540	0.89(0.46-1.74)	0.740	0.68(0.36-1.28)	0.229
Diabetes care team	0.33(0.16-0.66)	0.002	0.38(0.18-0.79)	0.010	0.70(0.37-1.33)	0.280
Other people living with diabetes	0.25(0.12-0.52)	0.000	0.33(0.16-0.69)	0.004	0.50(0.26-0.97)	0.040
Social media	0.24(0.12-0.48)	0.000	0.38(0.19-0.77)	0.008	0.75(0.39-1.41)	0.370
**Behaviour change (positive responses) (individual reference: No)**						
Check blood glucose more than usual	0.95(0.48-1.89)	0.889	0.73(0.35-1.47)	0.371	1.04(0.53-2.05)	0.906
More careful about taking medications than usual	0.52(0.21-1.28)	0.156	0.64(0.25-1.62)	0.347	1.33(0.58-3.06)	0.503
More exercise than usual	0.73(0.37-1.42)	0.353	0.87(0.43-1.74)	0.693	0.59(0.29-1.14)	0.116
Less exercise than usual	1.79(0.92-3.46)	0.082	1.82(0.91-3.63)	0.092	3.04(1.57-5.89)	0.001
Eat less than usual	0.63(0.33-1.21)	0.164	0.65(0.33-1.27)	0.205	1.34(0.71-2.53)	0.367
Eat more than usual	0.94(0.48-1.84)	0.847	1.03(0.51-2.09)	0.938	0.82(0.42-1.61)	0.571

Model was adjusted for age, sex, educational status and type of diabetes mellitus

## Discussion

After cases of COVID-19 were recorded in Ghana, there was a lot of fear, panic and tension among the entire citizenry, especially among the urban dwellers. The increased number of deaths of COVID-19 patients in China, Spain, USA, Brazil, etc. as reported and discussed by the media further heightened the fear and panic among Ghanaians especially when community spread of the infection began. The level of the emotional stress was more intense in people who were classified as a high-risk group including the aged, immunocompromised individuals and those with underlying chronic diseases such as diabetes, HIV, TB, cancer, etc. Therefore, this study sought to explore the psychosocial impacts of COVID-19 among people living with diabetes mellitus in Ghana.

Our findings showed that 33.8% of diabetes patients expressed worries about people with diabetes being characterized as a risk group (Figure 2). Similar to the findings in this study, an earlier study has demonstrated that diabetes patients may be overly worried about being described as a risk group during the COVID-19 pandemic [10]. Following the government of Ghana´s imposition of the restrictive measures to control the SARS-CoV-2, the health authorities intensified their education on the need for especially those with chronic illness including diabetes to adhere to the precautionary measures and remained quarantined at home because they had been categorized as a high-risk group and may exhibit severe symptoms of the pandemic if infected. However, the novelty of COVID-19 also means less scientific information and knowledge concerning the disease especially during the early phase of the pandemic thus culminating in the widespread of several misconceptions about the disease. The Danish Diabetes Association, for instance, reported earlier that only poorly controlled diabetes patients were at risk of COVID-19; but later declared that all people with diabetes mellitus, regardless of the type, regulation and complications, were classified as being at high risk [12]. The lack of knowledge on the novel COVID-19 was clear in Ghana where only 61.7% of the general population had some sort of knowledge about the outbreak [3], and about one-third of health care providers in Offinso-North District in Ghana had poor knowledge of the pandemic during the early stages [14]. The rise in morbidity and mortality of COVID-19, coupled with the significant lack of knowledge on the disease may upsurge the worries among individuals with diabetes and calls for the intensification of education and counseling on COVID-19.

Since the early COVID-19 outbreak in China, much attention has concentrated on people with diabetes mellitus because of poor prognosis in those infected with the disease. Preliminary reports were largely on people with type 2 diabetes mellitus, although current studies have shown that individuals with type 1 diabetes mellitus are also at risk of severe COVID-19. The cause for the worse prognosis in people with diabetes infected with the pandemic may be multifactorial; as age, sex, ethnicity, comorbidities such as hypertension and cardiovascular disease, obesity, a pro-inflammatory and pro-coagulable state contribute to the risk of worse outcomes [15]. Our study identified that “worries about being overly affected due to diabetes if infected with COVID-19” were seen more among age groups within 20-29 years. This could have been influenced by the increasing trend of COVID-19 infection among the younger population in seriously-hit countries especially the USA [16].

Our study revealed that the majority of the diabetic participants had developed complications including nephropathy, retinopathy, foot ulcer, heart disease and arthritis indicating the progression of diabetes (Figure 1). It is therefore not surprising to realize that most of the participants in the present study admitted being afraid and worried about contracting the COVID-19 infection, because of their anticipated fear of death. This finding is similar to earlier reports from a position statement from the American Diabetes Association on the psychological care for people with diabetes [9]. The study further affirmed that the presence of the contributing factors of diabetes complications influence the degree of distress and anxiety in these patients. Again, another study conducted by [17] which examined data on 1,561 patients with COVID-19 from two hospitals in Wuhan showed that those with diabetes were more likely to require admission to an intensive care unit (ICU) or to die. A similar cohort study involving 5,693 COVID-19 patients on admission in a hospital, revealed that the risk of death was more common in those with poorly controlled diabetes who also presented with progressive stages of the disease and other associated diseases [18].

Even though people with diabetes expressed a high sense of worry of being categorized as a risk group and not being able to manage diabetes if infected with COVID-19, the situation was more pronounced in females than males in our study, and this finding is synonymous with previous studies [10,19]. A study had reported a known history of diabetes as an independent predictor for morbidity and death in patients with SARS-CoV [20], and thus it was expected for the participants to be alarmed or worried during the present pandemic. Previous outbreaks such as SARS-CoV in 2002, Middle East Respiratory Syndrome (MERS-CoV) in 2012, and Ebola virus in 2014, generated fear and panic among citizens of affected countries, and this was prominent among people who were classified as high risk [21].

An effective measure to halt the community transmission of SARS-CoV-2 was to partially lock down certain densely populated areas in Ghana by the government, as well as quarantining suspected patients who exhibited classical symptoms of COVID-19 [22]. People, especially those with underlying illnesses such as diabetes, became overly anxious and developed moderate-high fear of being isolated when sick, as was identified in our study (Table 3). Compared with the general population, people with diabetes mostly felt an increased level of loneliness which was compounded during the COVID-19 pandemic [12]. People with diabetes exhibiting high levels of distress and feeling lonely would most likely escalate COVID-19 specific worries. The psychosocial effects of quarantine on people have been described in previous outbreaks; as this measure may cause confusion, anger as well as possible post-traumatic stress symptoms such as panic of being infected, dullness, frustrations, economic instability, stigma, insufficient supplies, etc [5,7].

We realized in our study that most of the diabetes patients received some significant forms of social support from relatives and friends, diabetic care team, other people living with diabetes, social media, workplaces, and schools (Table 4). Our study hence is contrary to another study that did not observe any association between the level of social support and COVID-19 specific distress [12]. However, it agrees with previous studies that have concluded that a lack of social network and social support contributes significantly to higher diabetes distress [23,24].

Our study was limited by the convenient sampling technique employed to select the participants. This might have introduced selection bias where patients with known COVID-19 specific distress may have accepted to participate. The current study therefore does not seek to generalize.

## Conclusion

This study reveals a sense of worry and anxiety among people living with diabetes during the current COVID-19 pandemic. The COVID-19 related worries in diabetes patients range from being part of a high-risk category, being overly affected due to diabetes if infected with COVID-19 and not being able to manage diabetes if infected with COVID-19. Continuous education and counseling on COVID-19 especially in persons with chronic infection are recommended to minimize the level of anxiety and emotional stress in these individuals. This study further reveals that during a pandemic, people with chronic conditions such as diabetes are likely to be concerned with how it impacts health possibly as a result of the potential disruptions to supply chain and services provisions. Thus, there is a need to intensify communication and offer reassurances to people with chronic conditions to alleviate anxiety and improve quality of life.

### What is known about this topic

- COVID-19 has presented with psychological problems including confusion, anger as well as possible post-traumatic stress symptoms such as panic of being infected, dullness, frustrations, economic instability, stigma and insufficient supplies;
- The aged, immunocompromised individuals and people with pre-existing diseases have been identified as risk groups.

### What this study adds

- This study establishes that people with chronic conditions such as diabetes are more likely to express worry due to, they referred to as risk group and due to the disruptions in service provisions due to lockdown restrictions.
